# Aortopulmonary Collateral Flow Is Related to Pulmonary Artery Size and Affects Ventricular Dimensions in Patients after the Fontan Procedure

**DOI:** 10.1371/journal.pone.0081684

**Published:** 2013-11-26

**Authors:** Heiner Latus, Kerstin Gummel, Tristan Diederichs, Anna Bauer, Stefan Rupp, Gunter Kerst, Christian Jux, Hakan Akintuerk, Dietmar Schranz, Christian Apitz

**Affiliations:** 1 Pediatric Heart Center, Justus-Liebig University Clinic, Giessen, Germany; 2 Department of Pediatric Cardiology, University Children’s Hospital Muenster, Muenster, Germany; Scuola Superiore Sant'Anna, Italy

## Abstract

**Background:**

Aortopulmonary collaterals (APCs) are frequently found in patients with a single-ventricle (SV) circulation. However, knowledge about the clinical significance of the systemic-to-pulmonary shunt flow in patients after the modified Fontan procedure and its potential causes is limited. Accordingly, the aim of our study was to detect and quantify APC flow using cardiovascular magnetic resonance (CMR) and assess its impact on SV volume and function as well as to evaluate the role of the size of the pulmonary arteries in regard to the development of APCs.

**Methods:**

60 patients (mean age 13.3 ± 6.8 years) after the Fontan procedure without patent tunnel fenestration underwent CMR as part of their routine clinical assessment that included ventricular functional analysis and flow measurements in the inferior vena cava (IVC), superior vena cava (SVC) and ascending aorta (Ao). APC flow was quantified using the systemic flow estimator: (Ao) - (IVC + SVC). Pulmonary artery index (Nakata index) was calculated as RPA + LPA area/body surface area using contrast enhanced MR angiography. The patient cohort was divided into two groups according to the median APC flow: group 1 < 0.495 l/min/m^2^ and group 2 > 0.495 l/min/m^2^.

**Results:**

Group 1 patients had significant smaller SV enddiastolic (71 ± 16 vs 87 ± 25 ml/m^2^; p=0.004) and endsystolic volumes (29 ± 11 vs 40 ± 21 ml/m^2^; p=0.02) whereas ejection fraction (59 ± 9 vs 56 ± 13%; p=0.38) differed not significantly. Interestingly, pulmonary artery size showed a significant inverse correlation with APC flow (r=-0.50, p=0.002).

**Conclusions:**

Volume load due to APC flow in Fontan patients affected SV dimensions, but did not result in an impairment of SV function. APC flow was related to small pulmonary artery size, suggesting that small pulmonary arteries represent a potential stimulus for the development of APCs.

## Introduction

Aortopulmonary collaterals (APCs) are frequently found in patients with a single-ventricle circulation at different stages of palliation [1]. These vessels typically originate from the subclavian arteries or their branches and represent additional sources of pulmonary blood flow that cause a left-to-right shunt with a subsequent volume load to the single-ventricle. 

Using standard CMR flow measurements, previous studies were able to reliably quantify the amount of APC flow in patients after bidirectional cavopulmonary connection (BCPC) and after the Fontan procedure [2-5]. Highlighting the clinical significance of APC flow, three studies recently demonstrated that increased collateral flow before the Fontan operation was associated with longer duration of pleural drainage and prolonged recovery in the postoperative period [6-8]. However, the mentioned studies primarily focused on technical aspects of quantifying APC flow [2-4] or were assessed in patients after the Glenn-procedure [6-8]. 

Data regarding the clinical implications of APCs in the midterm follow-up in patients with a total cavopulmonary connection (TCPC) is limited [4,9,10] but is of great interest as many centres routinely perform interventional closure of these collaterals. 

However, the effects of APC flow on blood flow distribution in the Fontan circulation and the pulmonary vascular bed is largely unknown. Therefore, the aim of our study was to quantify APC flow in Fontan patients and to assess its impact on clinical status and single ventricle dimensions and function, and pulmonary vascular hemodynamics. Furthermore, we sought to assess whether the pulmonary artery size does matter for the development of APCs.

## Materials and Methods

### Study population

The study included 60 consecutive patients with a Fontan circulation who underwent cardiac magnetic resonance imaging (CMR) examination as part of their routine clinical assessment between August 2008 and November 2012 at our institution. Candidates were excluded if: (1) CMR studies were incomplete or of poor image quality (e.g. due to metal or motion artefacts), (2) there was evidence of flow through a patent tunnel fenestration on echocardiography or CMR, (3) regurgitation at any valve was more than moderate on echocardiography, (4) CMR angiography revealed aortic coarctation.

Clinical data were retrospectively obtained from hospital medical records including date of birth, gender, anatomic diagnoses, age at and type of each surgical procedure, history of previous occlusion of aortopulmonary collaterals, age at CMR evaluation, laboratory findings (B-type natriuretic peptide) and data from cardiac catheterization.

The study protocol was approved by the University Medical Center Giessen review board and written consent for use of anonymised CMR and clinical data for research purposes was obtained from all patients or parents of the patients. 

### Cardiovascular magnetic resonance imaging (CMR)

#### Cine CMR

All CMR studies were performed on a 3-Tesla system (Verio, Siemens, Erlangen, Germany). Images were acquired in supine position with two sixteen-elements phased array coils. Sedation was applied in younger patients when considered necessary. The CMR protocol included a stack of short-axis slices from the base of the heart to the apex using cine steady-state free precession (SSFP) with breath-hold or gradient echo (GE) sequences in free-breathing technique when patients were sedated. 

Data acquired during breath-hold were assessed with the following sequence parameters: TR 48 ms, TE 1.5 ms, flip angle 60°, slice thickness 6 mm, in plane image resolution 1.3 mm x 1.3 mm x 6.0 mm, temporal resolution 25-40 phases. In measurements during free-breathing, the sequence parameters were as follows: TR 56 ms, TE 2.5 ms, flip angle 12°, slice thickness 5 mm, in plane image resolution 1.4 mm x 1.4 mm x 5.0 mm.

End-diastolic (maximal) and end-systolic (minimal) volume, stroke volume (SV) and ejection fractions (EF) for the single ventricle were calculated by dedicated software (ARGUS, Siemens, Erlangen, Germany) after the endocardial borders were traced manually at end-systole and end-diastole. All parameters were adjusted to body surface area (BSA). 

#### Flow measurements

Standard two-dimensional phase contrast flow measurements were used to quantify blood flow distribution in the Fontan circulation using a retrospectively gated gradient echo sequence during free breathing using the following parameters: TR 34 ms, TE 2.9 ms, flip angle 25°, slice thickness 5 mm, in plane image resolution 1.3 mm x 1.3 mm x 5.0 mm.

Flow measurements were performed perpendicular to each targeted vessel by the double oblique technique. Effective antegrade venous flow was measured in the superior vena cava (SVC) directly above the inflow into the Glenn anastomosis and below the insertion of the azygos vein. Flow measurements in the inferior vena cava (IVC) were performed at the level of the diaphragm. In patients with a bilateral cavopulmonary anastomosis, flow in both SVC’s was measured and the sum was quantified as SVC flow. The combined SVC and IVC flow volume was defined as total caval flow. In the ascending aorta, flow was measured just distal to the coronary arteries and the semilunar valves. In all cases, encoding velocity was adjusted to avoid aliasing. As described previously [2,3,9] APC flow was quantified as the difference between effective antegrade aortic flow and the sum of antegrade SVC and IVC blood flow as measured with phase contrast CMR (systemic flow estimator). 

When clinically indicated, cardiac catheterization was performed in the routine clinical fashion. The pulmonary vascular resistance (PVR) was calculated using the invasive pressure data and the CMR flows as the quotient between transpulmonary gradient and effective antegrade pulmonary flow defined as the sum of antegrade flow as measured in SVC and IVC plus collateral blood flow [8,10]. The transpulmonary gradient was calculated as the difference between mean pulmonary artery pressure and pulmonary capillary wedge pressure.

#### 3-dimensional contrast enhanced magnetic resonance angiography

A contrast-enhanced magnetic resonance angiographic (CE-MRA) scan was obtained in the coronal plane, using gadopentetate dimeglumine (Magnevist, Bayer, Leverkusen, Germany) at a dose of 0.15-0.2 mmol/kg and the following settings: repetition time 2.56 ms, echo time 0.94 ms, flip angle 19°, slice thickness 1.2 - 1.4 mm, 40 - 64 slices per slab, generalized autocalibrating partially parallel acquisition imaging, with an acceleration factor of 2. 

#### Image reconstruction and measurements

**Figure 1:**
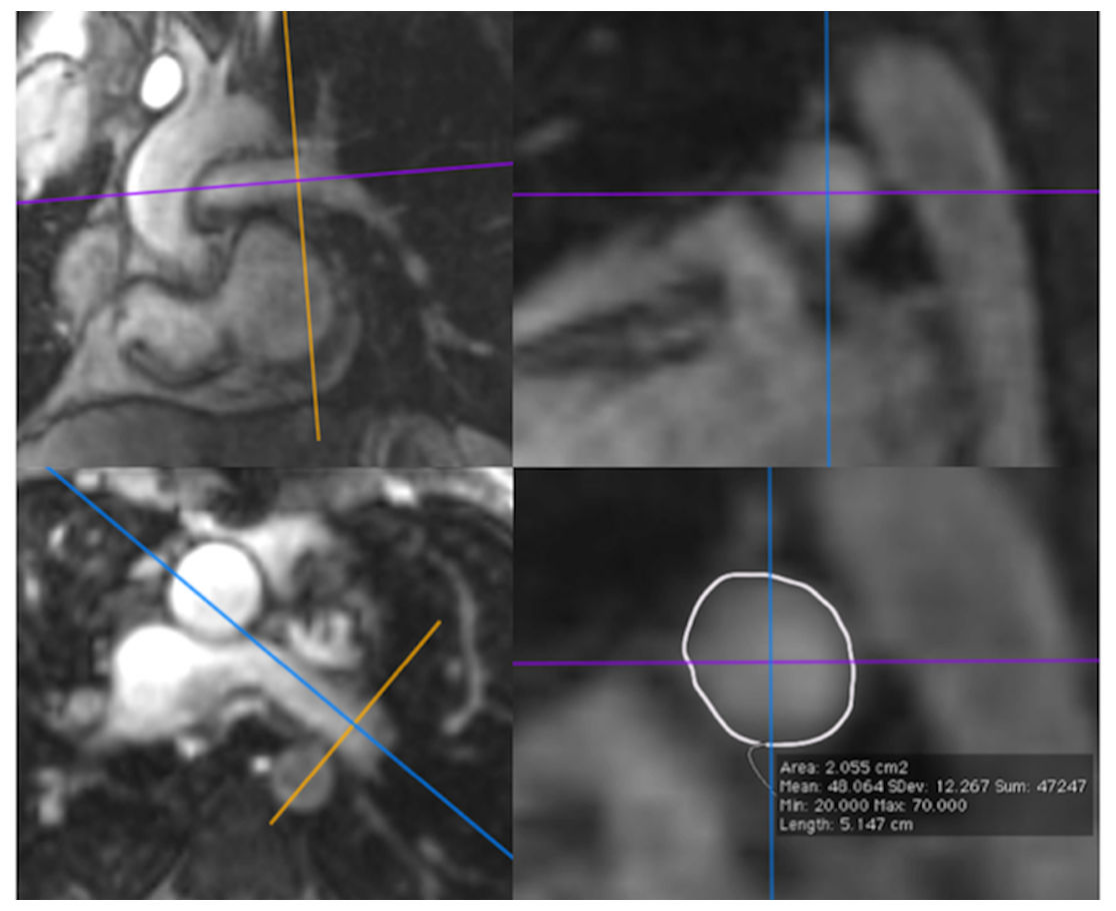
Multiplanar reformatted (MPR) images of the left pulmonary artery (LPA) obtained from contrast-enhanced magnetic resonance angiography (CE-MRA). Assessment of the size of the LPA is performed from the right images in a saggital plane.

CE-MRA data were reconstructed on off-line work station (OsiriX; OsiriX Foundation, Switzerland). User-defined maximum-intensity (MIP) projections and multiplanar reformatted (MPR) images were obtained using dedicated software (OsiriX Viewer). From the acquired sets of images, the one with the highest signal in the pulmonary arteries was chosen for reconstruction. The pulmonary artery index (i.e. Nakata index) was calculated as the sum of the cross-sectional areas of the left and right pulmonary artery divided by body surface area [11]. The areas were obtained from the reformatted angiographic images of the right and left pulmonary arteries (Figure **1**).

### Statistical analysis

All continuous variables were tested for normality using the Kolmogorov-Smirnov test and are presented as mean with standard deviation. Comparisons between groups were made with the Student t-test, the Mann-Whitney U test or the Fisher’s exact test, as appropriate. Pearson’s correlation coefficient was used to analyse simple linear relationships between different variables. 

A multiple regression analysis was performed to determine covariates associated with APC flow. The assumptions of linearity, independence of errors, homoscedasticity, unusual points and normality of residuals were met after a square-root transformation of the APC flow. Analysis was performed using GraphPad statistical software package (San Diego, California, USA) and R (Version 3.0.1) using the library car (Version 2.0-18). A *p* value ≤0.05 was considered statistically significant. 

## Results

### Patient characteristics

A total of 60 patients (22 females) after the Fontan procedure fulfilled the entry criteria and were enrolled in the study. Mean age at CMR examination was 13.3 ± 6.8 years (mean weight 42 ± 21 kg). To evaluate the impact of APC flow, the patient cohort was divided into two groups according to the median APC flow of 0.495 l/min/m^2^: patients with APC flow <0.495 l/min/m^2^ were assigned to group 1 while patients with APC flow ≥0.495 l/min/m^2^ were assigned to group 2 (Table **1**). 

**Table 1:** Demographic and clinical data of the study population as a whole and in the subgroups.

	Variable	All patients	APC flow < 0.495 l/min/m^2^	APC flow ≥ 0.495 l/min/m^2^	p Value
Demographic data	Patients, n	60	30	30	
	Male/Female	38/22	20/10	18/12	0.79
	Height , cm	144 ± 25	148 ± 21	141 ± 29	0.31
	Weight, kg	43 ± 21	44 ± 19	42 ± 23	0.71
	BSA (m^2^)	1.29 ± 0.43	1.32 ± 0.38	1.26 ± 0.48	0.56
	Single ventricle type				0.003
	Right, n (%)	24 (40)	6 (20)	18 (60)	
	Left, n (%)	35 (58)	23 (77)	12 (40)	
	Indeterminate, n (%)	1 (2)	1 (3)	0 (0)	
	Age at CMR study, years	13.3 ± 6.8	14.3 ± 7.2	12.3 ± 6.4	0.27
	Age at TCPC, years	4.1 ± 3.1	4.4 ± 3.8	3.7 ± 2.2	0.34
	Follow-up, years	9.2 ± 5.2	9.9 ± 5.0	8.6 ± 5.4	0.14
	Previous BCPC, n (%)	49 (82)	23 (77)	26 (87)	0.51
	Age at BCPC, years	1.5 ± 3.2	1.9 ± 4.4	1.3 ± 1.6	0.84
	Initial procedures				0.12
	APS, n (%)	34 (57)	19 (63)	15 (50)	
	cPAB, n (%)	8 (13)	3 (10)	5 (17)	
	PDA-Stent + bPAB, n (%)	10 (17)	2 (7)	8 (26)	
	PDA-Stent, n (%)	2 (3)	2 (7)	0 (0)	
	None, n (%)	6 (10)	4 (13)	2 (7)	
	Previous APC embolisation/coiling	4 (7)	1 (3)	3 (10)	0.61
	PA intervention, n (%)	16 (27)	4 (13)	12 (40)	0.039
	PA stent, n (%)	14 (23)	4 (13)	10 (33)	
	PA stenosis, n (%)	1 (2)	0 (0)	1 (3)	
	PA BAP, n (%)	1 (2)	0 (0)	1 (3)	
Clinical data	HR, bpm	77 ± 15	74 ± 13	80 ± 17	0.15
	SaO_2_, %	95 ± 3	96 ± 3	94 ± 4	0.044
	RR, mmHg	115/65	116/68	113/61	0.56
	NYHA-class, I/II/III/IV, n	24/34/2/0	12/18/0/0	12/16/2/0	0.58
	BNP, pg/ml (n=28)	28 ± 31	17 ± 10	38 ± 39	0.39

*APC*, aortopulmonary collateral flow*; BSA*, body surface area; *CMR*, cardiac magnetic resonance*; TCPC*, total cavopulmonary connectio*n; BCPC*, bidirectional cavopulmonary connection; *APS*, aortopulmonary shunt*; cPAB*, central pulmonary artery bandint; *bPAB*, bilateral pulmonary artery banding; *PDA*, persistent arterial duct; *PA*, pulmonary artery; *HR*, heart rate; *bpm*, beats per minute; *S*aO_*2*_, transcutaneous arterial oxygen saturation; *RR*, blood pressure; *NYHA*, New York Heart association; *BNP*, B-type natriuretic peptide; Data expressed as mean ± 1 standard deviation.

Age at study did not differ significantly between the two groups (14.3 ± 7.2 vs. 12.3 ± 6.4 years; p=0.29). Remarkably, the type of single-ventricle was unequally distributed: 77% of the patients in group 1 had a left systemic ventricle, 60% of the patients in group 2 had a morphologic right systemic ventricle (p=0.003). 

Initial procedures did not differ significantly between the two groups (p=0.12), albeit a combined approach with stenting of the arterial duct and placement of bilateral pulmonary artery banding had been performed only in 2 patients in group 1 (7%) but in 8 patients in group 2 (27%). 

77 % of group 1 and 87% of group 2 patients had previous volume unloading surgery by creation of a BCPC (p=0.51), while age at BCPC was similar in the two groups ((1.9 ± 4.4 years in group 1 and 1.3 ± 1.6 years in group 2; p=0.84). Fontan completion was performed at a mean age of 4.4 ± 3.8 years in group 1 and at an age of 3.7 ± 2.2 years in group 2 (p=0.34). Mean follow-up (time interval between TCPC and present CMR study) did not differ significantly between the two groups (9.9 ± 5.0 years in group 1 and 8.6 ± 5.4 years in group 2; p=0.14). 

### Clinical findings

There was no difference in the spectrum of clinical symptoms between both groups (p=0.58) (Table **1**). The majority of patients were in NYHA class I (40 vs 40 %) and II (60 vs 53 %), only 2 patients in group 2 were in class III (0 vs 7 %). Heart rate (74 ± 13 vs 80 ± 17 bpm; p=0.15) and blood pressure (116/67 vs 113/61 mmHg; p=0.56) were comparable in both groups. Transcutaneous oxygen saturations were significantly higher in group 1 patients (96 ± 3 vs 94 ± 4 %; p=0.04). Mean BNP-levels (available in 28 patients) were 28 ± 21 pg/ml (range 3 to 109 pg/ml) and did not differ significantly within the two groups (17 ± 10 vs 38 ± 39 pg/ml; p=0.39). 

### CMR findings

Patients in group 1 had significant smaller enddiastolic (71 ± 16 vs. 87 ± 25 ml/m^2^; p = 0.004) and endsystolic volumes (29 ± 11 vs. 40 ± 21 ml/m^2^; p=0.02) whereas ejection fraction (59 ± 9 vs. 56 ± 13%; p=0.38) showed no significant difference between the two groups (Table **2**). While SVC flow did not differ between the two groups (0.87 ± 0.27 vs. 0.92 ± 0.34 l/min/m^2^; p=0.50), IVC flow was significantly reduced in group 2 patients (1.68 ± 0.43 vs. 1.49 ± 0.56 l/min/m^2^; p=0.03). Total caval flow was similar in the two groups (2.55 ± 0.53 vs. 2.41 ± 0.27 l/min/m^2^; p=0.43) but there was a tendency towards an altered SVC/IVC blood flow ratio (0.54 ± 0.19 vs. 0.74 ± 0.72; p=0.21).

**Table 2:** CMR findings and invasive hemodynamic data of the study population.

	Variable	All patients	APC flow < 0.495 l/min/m^2^	APC flow ≥ 0.495 l/min/m^2^	p Value
CMR data	EDVi, ml/m^2^	79 ± 23	71 ± 16	87 ± 25	0.004
	ESVi, ml/m^2^	35 ± 17	29 ± 11	40 ± 21	0.02
	SVi, ml/m^2^	44 ± 11	41 ± 10	47 ± 12	0.04
	EF, %	58 ± 11	59 ± 9	56 ± 13	0.38
	SVC, l/min/m^2^	0.89 ± 0.30	0.87 ± 0.27	0.92 ± 0.34	0.50
	IVC, l/min/m^2^	1.59 ± 0.50	1.68 ± 0.43	1.49 ± 0.56	0.03
	Total caval flow, l/min/m^2^	2.48 ± 0.63	2.55 ± 0.53	2.41 ± 0.72	0.43
	AAO, l/min/m^2^	3.19 ± 0.88	2.80 ± 0.53	3.58 ± 0.99	<0.001
	APC, l/min/m^2^	0.71 ± 0.65	0.25 ± 0.16	1.17 ± 0.62	<0.0001
	SVC/IVC flow ratio	0.64 ± 0.53	0.54 ± 0.19	0.74 ± 0.72	0.21
	PAI (Nakata index), mm^2^/m^2^ (n=36)	319 ± 100	371 ± 86	256 ± 78	<0.001
Cath data	mPAP, mmHg	11 ± 3	11 ± 3	12 ± 4	0.55
(n=14)	TPG, mmHg	5 ± 1	5 ± 2	5 ± 1	0.81
	PVRI, WU/m^2^	1.8 ± 0.5	2.0 ± 0.6	1.6 ± 0.4	0.15

*APC, aortopulmonary collateral flow; BSA*, body surface area; *i*, indexed for BSA; *CMR*, cardiac magnetic resonance; *EDV*, enddiastolic volume; *ESV*, endsystolic volume; *SV*, stroke volume; *EF*, ejection fraction; *SVC*, superior vena vava; *IVC*, inferior vena cava; *AAO*, asecending Aorta; *PAI*, pulmonary artery index; *mPAP*, mean pulmonary artery pressure; *TPG*, transpulmonary gradient; *PVR*, pulmonary vascular resistance; Data expressed as mean ± 1 standard deviation.

In 36 patients, quantification of pulmonary artery size using CE-CMR was possible. 15 patients with former pulmonary artery procedures were excluded, while image quality was not sufficient in 2 and angiography was not performed in 6 patients. Group 1 patients had significant larger pulmonary artery index than patients in group 2 (371 ± 86 vs 256 ± 78 mm^2^/m^2^; p<0.001).

Invasive hemodynamic parameters were available in 14 patients (mean time interval between CMR and cardiac catheterization was 1.9 ± 1.8 months). There was no significant difference in mean pulmonary artery pressure (11 ± 3 vs 12 ± 4 mmHg; p=0.55), transpulmonary gradient (5 ± 2 vs 5 ± 1 mmHg; p=0.81) and indexed pulmonary vascular resistance (2.0 ± 0.6 vs 1.6 ± 0.4 WU/m^2^; p=0.15) between the two groups (Table **2**).

### Correlations

**Figure 2:**
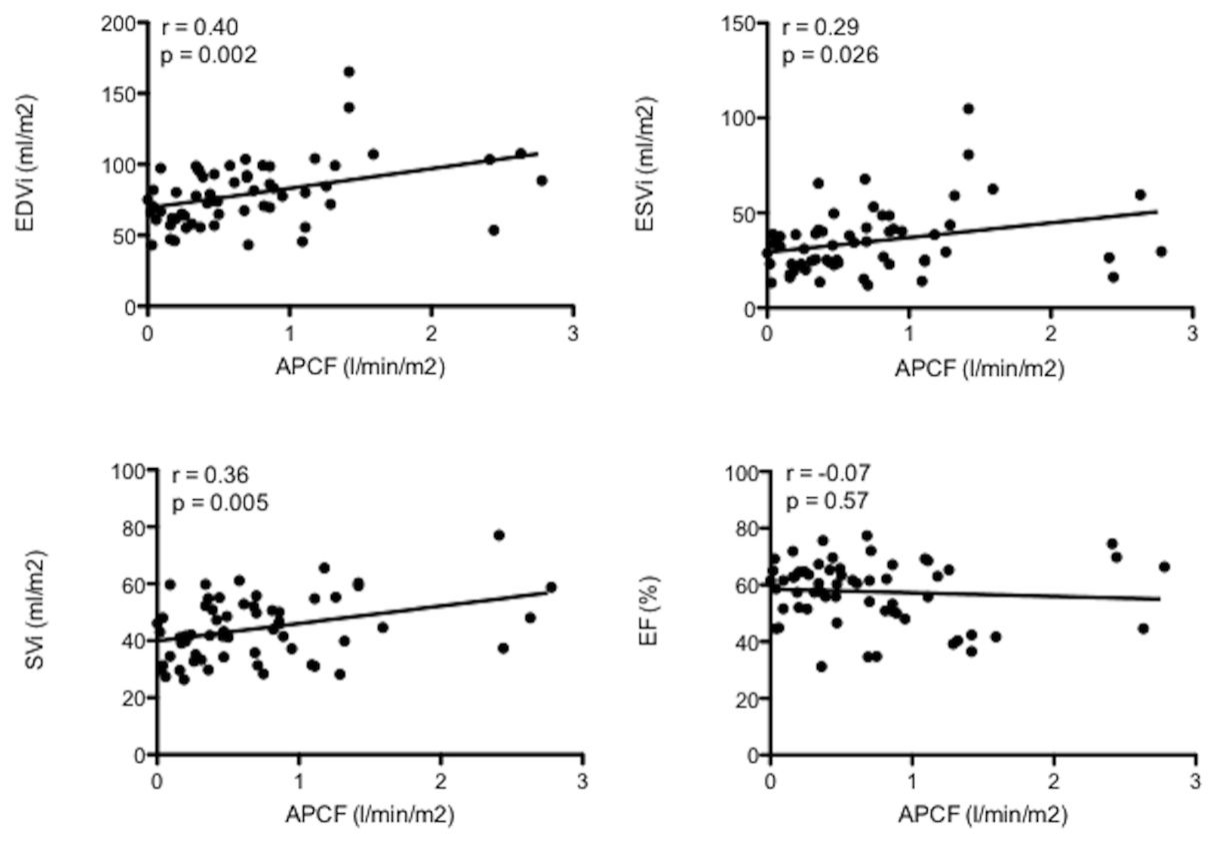
Significant relationships were found between APC flow and enddiastolic (r=0.40, p=0.002), endsystolic (r=0.29, p=0.026) and stroke volume (r=0.36, p=0.005) of the single ventricle (n = 60 patients). There was no relationship between the degree of APC flow and ventricular function expressed as ejection fraction (r=-0.07, p=0.57).

**Figure 3:**
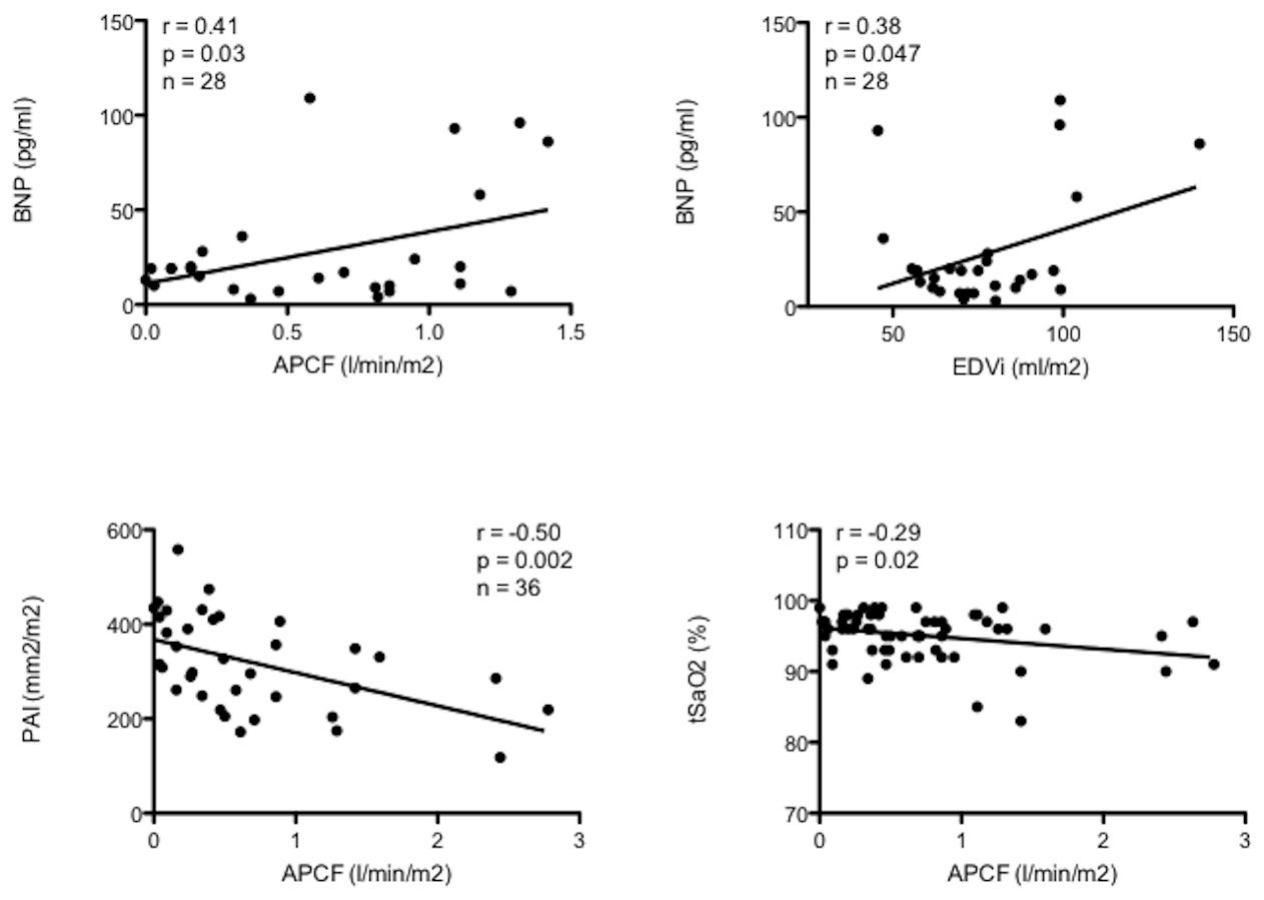
APC flow (r=0.41, p=0.03) and enddiastolic volume of the single-ventricle (r=0.38, p=0.047) showed a significant correlation with the levels of B-type natriuretic peptide (BNP) while pulmonary artery size (PAI) significantly affected the amount of APC flow (r=-0.50, p=0.002). A significant but weak correlation was found between the amount of APC flow and transcutaneous oxygen saturation (r=-0.29; p =0.02).

We found a significant relationship between APC flow and indexed enddiastolic (r=0.40, p=0.002), endsystolic (r=0.29, p=0.03) and stroke volume (r=0.36, p=0.005), but APC flow was unrelated to SV ejection fraction (r=-0.07, p=0.57) (Figure **2**). Levels of brain natriuretic peptide showed a significant relationship with the amount of APC flow (r=0.41, p=0.03) and with SV enddiastolic volume (r=0.38, p=0.05) (Figure **3**) but no relation to SV ejection fraction (r= 0.11, p=0.57). Pulmonary artery size (Nakata index) showed a significant inverse correlation with APC flow (r=-0.50, p=0.002) (Figure **3**). Transcutaneous oxygen saturations showed a weak but significant inverse relation with APC flow (r=-0.29, p=0.02) (Figure **3**).

There was no relation between APC flow and age at BCPC- (r=0.08, p=0.60), age at TCPC-operation (r=0.12, p=0.38) or follow-up since TCPC-operation (r=0.04, p=0.77).

**Figure 4:**
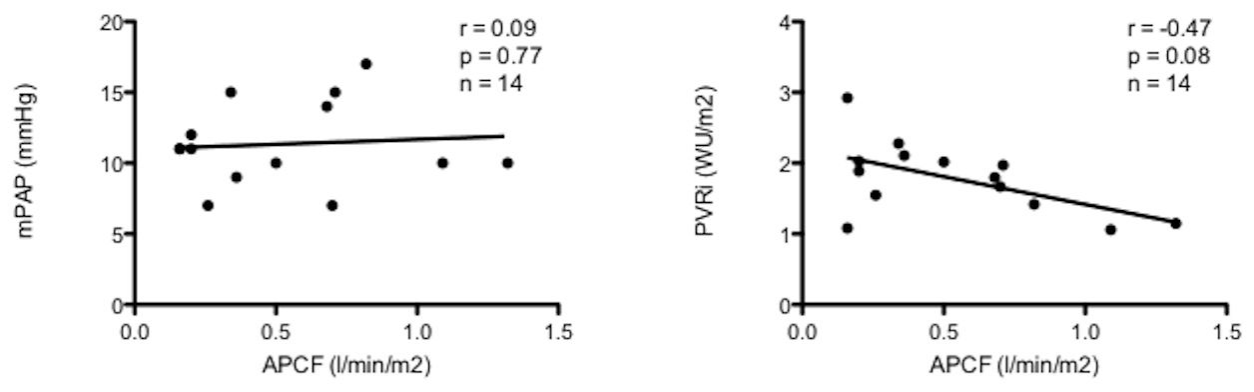
No correlation was found between APC flow and mean pulmonary artery pressure (r=0.09, p=0.77) but tendency (r=-0.47, p=0.08) towards lower pulmonary vascular resistance in the presence of increased APC flow.

Mean pulmonary artery pressures did not correlate with APC flow (r=0.09, p=0.77) but there was a tendency towards lower pulmonary vascular resistance in the presence of increased collateral flow (r=-0.47, p=0.08) (Figure **4**).

### Predictors of APC flow

Multiple regression analysis was performed to predict APC flow from pulmonary artery size, transcutaneous oxygen saturation and systemic ventricle. These predictor variables demonstrated their qualification for inclusion in the model by univariate associations with APC flow with p<0.1. These variables predicted APC flow (p<0.001; R^2^ = 0.35). While pulmonary artery size added statistically significantly to the prediction (p<0.05), transcutaneous oxygen saturation (p=0.07) and systemic ventricle (p=0.08) showed a weak statistical significance. 

## Discussion

In the presented study we were able to demonstrate that the presence of APC flow in SV patients after the modified Fontan procedure affected SV dimensions, due to volume overload by the left-to-right shunt, while the systolic function of the SV was unimpaired. Remarkably, APC flow was related to smaller pulmonary artery size, suggesting that small pulmonary arteries may represent a stimulus for the development of APCs in Fontan patients, a phenomenon that has been previously discussed in patients after the Glenn-operation [12], but has never been described in Fontan patients. 

When being detected by cardiac catheterization, many centres routinely perform interventional embolization of APCs in SV patients [13]. According to our results, APC embolization alone in the presence of small pulmonary arteries would only treat the symptom and not the cause. While interventional occlusion of the APCs would reduce left-to-right shunting that subsequently causes volume overload of the single-ventricle, this intervention alone does not eliminate the stimulus of collateral formation unless being combined with consequent treatment of pulmonary artery narrowing or stenosis as well as aortic arch obstructions if present. Dori et al. assessed the acute effects of interventional closure of APCs on flow hemodynamics in BCPC patients and revealed a significant decrease in APC flow and an increased blood flow in the SVC and the systemic circulation [14]. However the long-term effect of APC coiling or embolization alone needs to be assessed and should be compared with a combined approach with APC coiling and relieve of PA stenosis. 

Indeed, among multiple factors that have been suggested to lead to the formation of APCs, branch pulmonary artery stenosis as well as diminished or abnormal blood flow pattern in the pulmonary arteries have been considered as stimulus for the development of APCs [6,9]. Furthermore, besides the native structure and size of the pulmonary arteries, the effect of the initial procedures needs to be considered. The impact of aortopulmonary shunt procedures as well as the potential effects of a bilateral pulmonary artery banding on pulmonary artery growth, distorsion and reintervention rates needs to be investigated in further studies. Recently, Baba and colleagues compared Norwood- and Hybrid-procedure in the palliation of hypoplastic left heart syndrome (HLHS) and found significant differences in re-intervention rates and pulmonary artery size at the time of BCPC [15]. Our data showed a similar trend as a combined approach with stenting of the arterial duct and placement of bilateral pulmonary artery banding had been performed only in 2 patients in group 1 but in 8 patients in group 2, a result that suggests an increased presence of APCs in patients after the hybrid procedure while not being significant due to the small sample size in this subpopulation. Further studies with larger patient numbers are clearly required to study this phenomenon.

In accordance with the findings of Prakash and colleagues who revealed HLHS as a risk factor for increased APC flow, we found significant more patients with a systemic right ventricle in the group with high APC flow. Whether this is a specific long-term complication of the right ventricle in systemic position still remains unknown although specific risk factors for the development of APCs have already been identified in children with HLHS [16].

Although APC flow caused ventricular dilatation, myocardial function was unaffected by the additional volume overload. These results indicate that SV dysfunction seems relatively unrelated to volume overload but is probably more affected by afterload conditions, as previously described [17-19]. Based on our results and current studies that investigated the effect of additional volume overload through aortopulmonary shunt implantation in BCPC-patients [20,21], it is unlikely that a mild or moderate amount of APC flow leads to myocardial dysfunction. However, a cut-off value for volume overload needs to be determined, that allows an optimal ventricular-arterial coupling by matching pre- and afterload to maintain efficient myocardial performance in these patients [19].

In the presented patient cohort, the median APC flow was 0.495 l/min/m^2^, which is comparable to the findings of Prakash et al. [9] who measured a median shunt flow of 0.43 l/min/m^2^. Previous studies revealed a higher prevalence [1] and a higher degree of APC flow at the stage of a BCPC with a subsequent decline in shunt-flow after establishment of a total cavopulmonary connection (TCPC) [9]. The authors suggested that oxygen desaturation represents a potential stimulus for APC development and that abolishment of cyanosis after TCPC-completion leads to regression of the collaterals. Interestingly, we were also able to find lower transcutaneous oxygen saturations in the patient group with more APC flow, even if the observed lower oxygen saturations in some patients could theoretically also be related to diminutive venovenous collaterals that were not detectable on MR angiography. 

### APC flow and levels of B-type natriuretic peptide

The mean BNP values of 28 pg/ml in our study population match with previous studies that found BNP values of asymptomatic Fontan patients were comparable to those of healthy age-matched controls [22-24]. Regarding the utility of BNP in single-ventricle patients these studies showed somewhat inconsistent results. While in some studies higher BNP-levels were associated with type of TCPC-connection, degree of atrioventricular valve regurgitation, ventricular morphology, diastolic function, and clinical status [22-26], the use of BNP as an early marker of ventricular dysfunction, a surveillance tool or as an endpoint in clinical studies remains questionable [22,27].

Our study provided new data regarding the value of BNP-levels in single-ventricle patients. Despite unloading of the ventricle after completion of the Fontan circulation, the relationship between the amount of APC flow and ventricular dilatation with the observed rise in BNP-levels demonstrates the hemodynamic relevance of the additional volume overload. As reported previously [26], we found no relation between BNP and systolic SV function. Therefore, in Fontan patients with preserved myocardial function and no other valvular lesions, BNP-levels appear to relate rather with ventricular volume overload due to APC shunt flow.

### Effect of APC flow on pulmonary vascular hemodynamics

APCs supply systemic arterial blood to the distal pulmonary vasculature where it competes with blood flow from the pulmonary arterial system. Theoretically, chronic elevated shunt flow to the lung creates areas of high-pressure and increased pulmonary vascular resistance [28,29] and therefore has the potential to contribute to adverse clinical outcomes. On the other side, APC flow promotes pulsatile flow to the pulmonary vessels, thereby increasing shear stress on the endothelium that consecutively releases more nitric oxide, a potent vasodilator, that subsequently lowers pulmonary vascular resistance. In our subgroup analysis of 14 patients of whom invasive hemodynamic data were available, there was no significant difference in mean pulmonary artery pressure or pulmonary vascular resistance between the two groups. 

The observed inverse relationship between APC flow and vascular resistance indicates that additional pulsatile flow through APCs into the pulmonary vasculature seems not to increase, but even lowers pulmonary vascular resistance [10]. However, it has to be kept in mind that the mean follow-up after Fontan procedure was below 10 years and the long-term effect of these collaterals on the structure and remodelling of the distal pulmonary vascular bed remains unknown. It appears possible that the chronic shear stress may result in endothelial dysfunction and increased pulmonary vascular resistance in the long-term. 

Regarding flow in the TCPC-circulation, patients with a greater extent of APC flow showed significantly reduced IVC flow while SVC flow and total caval flow did not differ in the two groups. As a consequence, we observed that the flow volume distribution between the SVC and the IVC, which normally is about 1:2 [5], was abnormal in patients with more APC flow. Ovroutski et al. found decreased IVC flow and altered SVC flow ratio to be related to suboptimal hemodynamic outcome in Fontan patients [30]. Therefore, APC flow seems to impair subdiaphragmatic venous return in the Fontan pathway probably by competing with pulmonary blood flow. As the patients in our study were predominantly in NYHA functional classes 1 or 2, we can only speculate about the impact of APCs in patients with a “failing” Fontan circulation.

### Study limitations

Although standard CMR flow measurements represent a reliable way of estimating APC flow, there exists no reference technique to measure systemic-to-pulmonary shunt flow in patients with a single-ventricle circulation. Furthermore, standard values of single ventricle volumes do not exist so far. Therefore, we chose to compare a relatively homogenous patient population. Patients with a patent fenestration or with significant valvular lesions were excluded which lead to a certain selection bias. The observed inverse relationship between APC flow on PVR must therefore be related to the selective patient group analysed and does not allow any conclusions with regards to long-term impact of APC’s on the pulmonary vasculature. This study was designed as a mid-term, cross-sectional study, and was not designed to assess the effects of APC flow on long-term outcome in the single-ventricle population. 

## Conclusions

In conclusion, volume load due to APC flow in Fontan patients affects SV dimensions, but does not result in an impairment of SV function. Small pulmonary artery size appears to represent a stimulus for the development of APCs. Further studies are needed to reveal the prognostic relevance of APCs and to assess the impact of interventional closure of APCs on pulmonary vascular remodelling.
